# Development and Characterization of 15 Novel Genomic SSRs for *Viburnum farreri*

**DOI:** 10.3390/plants10030487

**Published:** 2021-03-05

**Authors:** Trinity P. Hamm, Marcin Nowicki, Sarah L. Boggess, William E. Klingeman, Denita Hadziabdic, Matthew L. Huff, Margaret E. Staton, Robert N. Trigiano

**Affiliations:** 1 Department of Entomology and Plant Pathology, University of Tennessee, Knoxville, TN 37996-4560, USA; thamm1@vols.utk.edu (T.P.H.); mnowicki@utk.edu (M.N.); sbogges1@vols.utk.edu (S.L.B.); dhadziab@utk.edu (D.H.); mhuff10@utk.edu (M.L.H.); mstaton1@utk.edu (M.E.S.); 2 Department of Plant Sciences, University of Tennessee, Knoxville, TN 37996-4561, USA; wklingem@utk.edu

**Keywords:** microsatellite, Adoxaceae, genetic diversity, gSSR

## Abstract

The *Viburnum* genus is of particular interest to horticulturalists, phylogeneticists, and biogeographers. Despite its popularity, there are few existing molecular markers to investigate genetic diversity in this large genus, which includes over 160 species. There are also few polymorphic molecular tools that can delineate closely related species within the genus. *Viburnum farreri*, a member of the *Solenotinus* subclade and one of the centers of diversity for *Viburnum*, was selected for DNA sequencing and development of genomic simple sequence repeats (gSSRs). In this study, 15 polymorphic gSSRs were developed and characterized for a collection of 19 *V. farreri* samples. Number of alleles per locus ranged from two- to- eight and nine loci had four or more alleles. Observed heterozygosity ranged from 0 to 0.84 and expected heterozygosity ranged from 0.10 to 0.80 for the 15 loci. Shannon diversity index values across these loci ranged from 0.21 to 1.62. The markers developed in this study add to the existing molecular toolkit for the genus and will be used in future studies investigating cross-transferability, genetic variation, and species and cultivar delimitation in the *Viburnum* genus and closely allied genera in the Adoxaceae and Caprifoliaceae.

## 1. Introduction

The genus *Viburnum* L. (Adoxaceae, formally classified in Caprifoliaceae [1]) includes about 163 species [2] that are native to temperate and subtropical regions of the Northern Hemisphere and extend into the mountains of South America and Asia [3,4]. Major centers of species diversity occur in eastern Asia and Latin America [5,6,7,8]. Species of *Viburnum* range from shrubs to small trees, and there are more than 70 species and interspecific hybrids in cultivation [9]. In 2017, Viburnums generated USD23.2 million in wholesale and retail sales in the U.S. alone [10]. In addition to horticultural value, the *Viburnum* genus also serves as a suitable model for studying phylogeography and evolution [2,4,11,12,13,14]. Much progress has been made in the phylogenetic classification of this genus [4,15,16,17,18,19] ranging from moving *Viburnum* and related genera from Caprifoliaceae to Adoxaceae [20] to providing formal phylogenetic definitions for 30 clades and subclades within *Viburnum* [21].

*Viburnum farreri* Stearn, fragrant viburnum, is classified within the clade *Crenotinus* and the subclade *Solenotinus* [21] and is native to the East Asian center of diversity. This China-native species produces paniculate inflorescences with opposite branches [22], making cultivars such as ‘Album’, ‘Nanum’, and ‘Candidissimum’ popular ornamental specimens. *Viburnum farreri*, historically known as *V. fragrans*, was first introduced to European gardens in 1911 [23]. Despite a rich history of cultivation, molecular tools are not available for determining genetic diversity and population structure of this species or other species in the *Solenotinus* subclade. Development of resources that could be used to delineate species and investigate genetic diversity within *Viburnum* would assist breeding programs and help resolve phylogenetic topology at low taxonomic levels. Attempts have been made to use barcode sequences to distinguish species, but due to inadequate sequence diversity, this method had limited applicability in differentiating species within subclades [17]. Recent literature has discussed the need to revisit the taxonomy of several *Viburnum* subclades, including *Solenotinus*, to more definitively draw conclusions on the evolutionary history of the genus [2,13,24].

Microsatellites, also known as simple sequence repeats (SSRs), are tandem repeats located throughout most eukaryotic genomes, which can be composed of mono-, di- to penta-nucleotide motifs [25]. They are a class of neutral markers that are co-dominant in nature. SSRs are particularly suited to study closely related individuals and species due in part to their highly polymorphic nature, caused by polymerase slippage during DNA replication [26]. Flanking regions of these repetitive motifs are mostly conserved, which allows for designed primers to target amplification of the SSR loci [27]. The resulting polymerase chain reaction (PCR) products can then be sized to determine the polymorphisms and therefore, alleles of specific loci. As a result of these properties, SSRs are frequently used to investigate genetic diversity [28,29] and delimit species and subspecies [30,31].

Historically, SSR markers have been developed by constructing microsatellite libraries. SSRs are now commonly discovered by mining next generation sequencing (NGS) data. Genomic SSR markers (gSSRs) are developed from genomic sequences, whereas expressed sequence tag SSR markers (EST-SSRs or eSSRs) are developed from RNA sequencing data. gSSR typically exhibit more alleles and are more informative for genotyping and estimating genetic diversity compared to eSSRs [32,33]. SSR markers in general can cross-transfer to closely related species and genera to yield informative products [34]. eSSRs commonly display greater cross-transferability than gSSRs because they are more likely to be within functional gene sequences and therefore are typically more conserved [35,36]. eSSRs and gSSRs have both transferred to closely related species within and outside of their genus, including well-studied economically important agronomic crops [34,37] as well as a number of woody ornamental species including *Cercis canadensis* [38], *Cornus* species [39], and *Fothergilla* species [40].

gSSRs have been developed for four of the ~163 species of *Viburnum* including *V. dilatatum* [41], a member of the *Succotinus* subclade, *V. rufidulum* [42], a member of the *Lentago* subclade, and *V. triphyllum* and *V. pichinchense* [43], which are members of the *Oreinotinus* subclade [21]. These gSSRs were developed for species/cultivar identification as well as investigation of population genetic diversity. They represent species in three of the larger clades (*Laminotinus, Valvatotinus*, and *Porphyrotinus*, respectively), leaving only *Crenotinus* unrepresented. A preliminary study on the cross-transferability of the markers developed for *V. dilatatum* [41] and *V. rufidulum* [42] was conducted and demonstrated wide, but not complete transferability of these gSSRs [44]. Inclusion of markers for *V. farreri,* a member of the *Crenotinus* clade and the *Solenotinus* subclade, would provide more extensive coverage of species across the entire *Viburnum* genus.

The objectives of this study were to develop additional gSSRs from de novo assembled genomic Illumina sequencing data of *V. farreri* ‘Nanum’ and apply them to estimate the genetic diversity of the species. The markers described herein from *V. farreri* in the *Solenotinus* subclade make the overall set of developed markers for the genus more complete and will allow study of cross-transferability to all subclades as described by Clement et al. [21] for use in downstream studies of the *Viburnum* genus and other closely related genera.

## 2. Results and Discussion

### 2.1. gSSR Development

**Figure 1:**
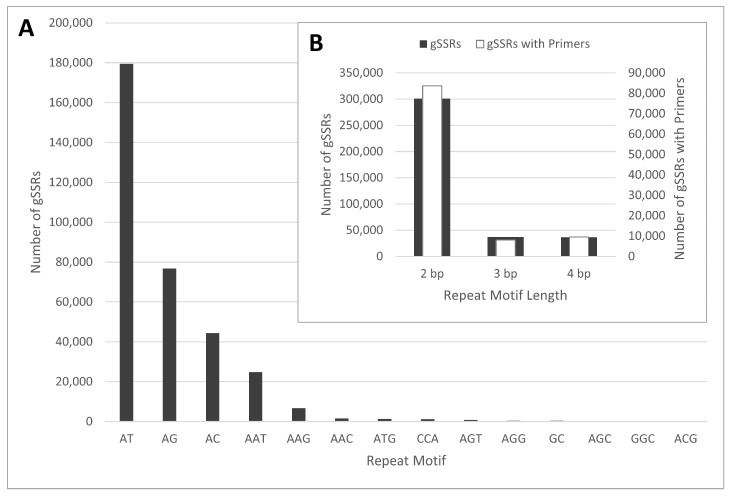
Genomic simple sequence repeats (gSSRs) discovered in the de novo assembled genome of *Viburnum farreri* ‘Nanum’. Overall number of gSSRs identified with our algorithm are in grey, based on repeat motif (**A**) and repeat motif length (**B**). Note specific repeat motif frequencies for tetra-nucleotide repeats were not calculated and therefore not included in (**A**). The number of gSSRs with primers designed for the locus based on repeat motif length are depicted in white and on the secondary axis (**B**). bp = base pairs.

A total of 14,541,259 assembled scaffolds of *V. farreri* ‘Nanum’ were analyzed for gSSRs with a minimum of six repeats for di- and tri-nucleotide motifs and a minimum of four repeats for tetra-nucleotide motifs. gSSRs were located in 390,541 of the scaffolds with a total of 424,029 SSRs identified and included the following: 301,148 di-, 37,171 tri-, and 36,696 tetra-nucleotide repeats (Figure 1). [AT]_n_ was the most (179,546 SSRs) commonly found motif. These results are similar to other gSSR studies developed from NGS data, including [AT]_n_ as the most common di-nucleotide repeat and [GC]_n_ being the least common [45,46]. Primer pairs were developed for 101,174 SSRs including 83,687 di-, 7996 tri-, and 9491 tetra-nucleotide repeats. Primers were not developed for any of the 49,014 identified compound SSRs (defined as SSRs separated by less than 15 bp). Fifty primer pairs were selected for the initial screening, and 15 were informative for our *V. farreri* collection.

### 2.2. gSSR Characteristics and Diversity Indices

Sixty-seven alleles were detected with the 15 gSSRs and loci yielded two- to- eight alleles per locus with an average of 4.47 alleles per locus (Table 1). Selected, polymorphic gSSRs present promising resources that can be used to assess genetic diversity within larger datasets. These gSSRs could also potentially be used to identify cultivars because they were able to capture genetic variability among the studied cultivars. The percent of missing data per locus ranged from 0 to 21 with only 3.9% missing data in the entire dataset. The only missing data were with samples from herbaria that were collected between 1966 and 1987. Therefore, the missing data could be due to low quality DNA and not mutations in the primer regions. Furthermore and more notably, nine out of the 15 gSSRs were not missing any data. The observed heterozygosity (H_o_ = 0.23) varied greatly from the expected heterozygosity (H_e_ = 0.60), and this result may be explained by the cultivated origin of most of the samples. The Shannon Diversity Index was very low (1.10) and indicated low allele species richness/evenness, which could be caused by the limited number of samples used in this dataset. These initial values of basic diversity measures are included as an illustration and possible point of reference for future in-depth studies of this (or related) species.

**Figure 2:**
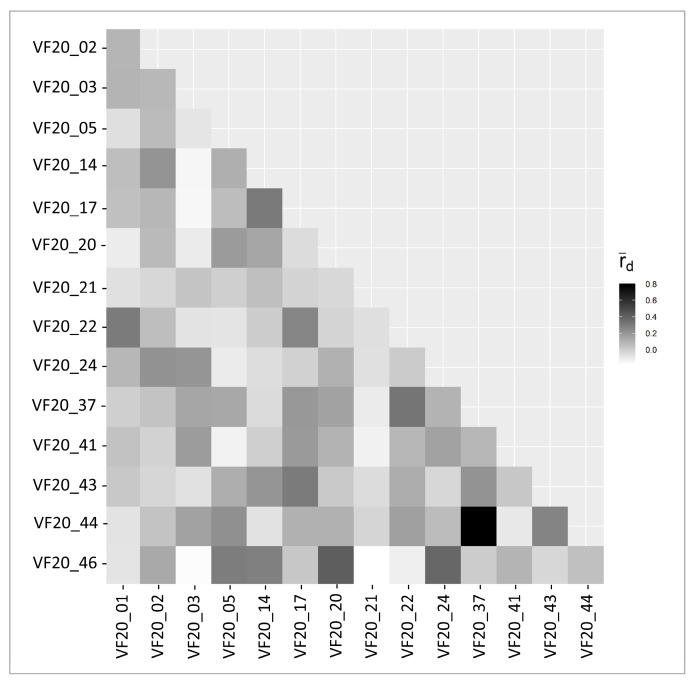
Linkage disequilibrium of the developed genomic simple sequence repeats (gSSRs). Pairwise ${\bar{r}}_{d}$ was calculated and displayed in a heatmap to infer if any loci were associated. The darker the square, the more of an association there is between the two loci. The lighter the square, the less of an association.

Linkage disequilibrium among loci was investigated using the standardized index of association (${\bar{r}}_{d}$), which accounts for the number of loci sampled [47]. The pairwise comparison between loci revealed a range of ${\bar{r}}_{d}$ from −0.18 to 0.80 (Figure 2), but the only loci with a high pairwise ${\bar{r}}_{d}$ were VF20_37 and VF20_44. Therefore, VF20_37 and VF20_44 were in linkage disequilibrium. Most loci used in this study were well-dispersed throughout the *V. farreri* genome because most pairs of loci did not have a high ${\bar{r}}_{d}$. The linkage disequilibrium observed between VF20_37 and VF20_44 can be caused by numerous factors including, but not limited to, physical proximity. Other factors that could have contributed are population differentiation, asexual reproduction, and natural selection [47]. It is crucial to demonstrate the SSRs are generally lacking any possible pairwise linkage. Indeed, were more, or stronger linkages detected, those would render our markers useless, as this would skew any diversity measures relying on markers undergoing independent inheritance. As no genomes are available for Dipsacales, we are unable to verify whether the single pair of SSRs showing LD is indeed in physical proximity to one another.

The studied dataset was small and does not consist of individuals within the same population. All accessions were obtained from arboreta and herbaria, and all individuals except one were cultivated and/or of unknown origin. Recent wild-collections of *V. farreri* are very scant and made obtaining native samples and samples in general difficult. Many cultivated plants are the product of non-random mating and clonal reproduction, which may have contributed to the two markers being in linkage disequilibrium. Consequently, the VF20_37 and VF20_44 gSSRs may not be physically close or linked to each other in the genome. More samples would be needed to investigate the linkage disequilibrium further. Although two of the 15 gSSRs developed were associated, this will not diminish the utility of this marker set as a resource for studying genetic diversity in the species.

Previously, gSSRs have been used successfully in cross-transferability studies with ornamental plant genera including *Cercis* (redbud) [38] and *Cornus* (dogwood) [39]. gSSRs were also developed for safflower with similar methods to our study, and those markers also displayed cross-transferability [48]. A preliminary study of cross-transferability of *Viburnum* species gSSRs developed in [41,42] was completed and indicated wide-transferability was possible [44]. Therefore, the markers developed in this study should have some cross-transfer success with other *Viburnum* species and help fill in missing data from gaps left by the previously developed markers. In future studies, we plan to explicitly evaluate the cross-transferability of these markers to other *Viburnum* species, other species in the Adoxaceae, as well as related genera in the Caprifoliaceae, such as *Lonicera* and *Weigela*.

## 3. Materials and Methods

### 3.1. Plant Materials and gDNA Extraction

Samples of *V. farreri* were obtained from the Morton Arboretum (MA), Mt. Airy Arboretum, Arnold Arboretum (AA), University of Washington Botanical Garden (UWBG), U.S. National Arboretum (USNA), and U.S. National Arboretum Herbarium (NA) (Table 2). Nineteen of the 22 samples were of garden origin (Table 2). Living specimens sampled from arboreta were originally collected and planted between 1940 and 2020, and herbarium specimens were collected between 1938 and 2007.

Leaves from arboreta samples were dried before DNA extraction, except for the *V. farreri* ‘Nanum’ (collected from the Mountain Hort. Crops Res. & Ext. Center at North Carolina State University) sample, which was flash frozen and used for Illumina MiSeq sequencing. DNA was sequenced in order to maintain a similar discovery method to all other SSRs designed for *Viburnum*. All samples were frozen in liquid nitrogen and then homogenized once, or twice if needed, using a Beadmill 24 (Fishers Scientific, Pittsburgh, PA, USA). Genomic DNA (gDNA) was isolated from the ‘Nanum’ sample with a CTAB method [49]. gDNA was isolated from all other samples using an Omega E.Z.N.A. Plant DNA Kit (Omega Bio-tek Inc., Norcross, GA, USA). The manufacturer’s protocol was followed except that 2% polyvinylpyrrolidone (PVP) (Fisher BioReagents, Waltham, MA, USA) was added to the P1 Buffer and the incubation time at 65 °C was increased from 10 min to 30 min. Quality of the isolated gDNA was assessed with a NanoDrop Lite Spectrophotometer (Thermo Fisher Scientific, Waltham, MA, USA). If gDNA isolated from samples with the E.Z.N.A. kit was unsuccessful in downstream applications, gDNA was re-isolated from those samples using the CTAB method [50].

### 3.2. gSSR Development and Screening

gDNA extracted from the ‘Nanum’ sample was submitted for Illumina MiSeq 600v3 (paired-end 2 × 300 bp) sequencing [Oklahoma Medical Research Foundation (OMRF), Oklahoma City, OK, USA]. The miSeq raw reads are available at NCBI Bioproject PRJNA706016. Read quality was assessed with FastQC version 0.11.7 [51] before and after trimming and quality filtering with Trimmomatic version 0.39 [52]. The minimum read length kept was set to 36 base pairs (bp). Reads were only retained if the mean quality score was ≥30. Reads were trimmed of adapter sequences and ends were trimmed until a minimum q-score of 30 was reached. The reads were assembled using Assembly By Short Sequences (ABySS) version 2.1.4 [53]. Default settings were used outside of the k-mer size, which was set to 64. DustMasker version 2.10.0 [54] was used to mask low-complexity DNA sequences before mining for SSRs. Finally, the masked file and assembled scaffolds were inputted into a custom Perl script [55] to identify SSR regions and develop primers with Primer3 version 2.5.0 [56]. This script searched for di-, tri-, and tetra-nucleotide repeats with designed primers that would result in a product size between 100 and 400 bp.

Twenty di-, 15 tri-, and 15 tetra-nucleotide primer pairs (total of 50) were selected randomly from outputs of the Perl script for screening Primers were synthesized by Integrated DNA Technologies (IDT, Coralville, IA, USA). PCR was conducted with all 50 primer pairs on three randomly selected *V. farreri* gDNA samples. The reaction volume was 10 µL and included 5 µL of 2× AccuStart II PCR SuperMix (Quantabio, Qiagen Beverly, Inc., Beverly, MA, USA), 3 µL autoclaved water, 1 µL of mixture of 5 µM forward and reverse primers, and 1 µL of 2 ng/µL of gDNA. The PCR thermal profile included 3 min of initial denaturation at 94 °C, 10 touchdown [57] cycles (94 °C for 40 s, 63 °C −0.5 °C/cycle for 40 s, and 72 °C for 30 s), 30 cycles (94 °C for 40 s, 58 °C for 40 s, and 72 °C for 30 s), and a final extension of 4 min. The PCR products were visualized with capillary electrophoresis (QIAxcel Advanced Electrophoresis System; Qiagen) and analyzed using a 25 to 500 bp DNA size marker and an internal 15/600 bp alignment marker. Of the 50 primer pairs evaluated, 17 had well-defined peaks and were polymorphic; other primer pairs can be investigated in future studies. These 17 primer pairs were used to amplify DNA from the 22 samples. Two *Viburnum* samples and two primer pairs were eliminated from the study because of low and/or inconsistent amplification. The allele sizes were determined using QIAxcel ScreenGel version 1.6.0.10. Raw allele sizes were then statistically binned into allelic classes with FlexiBin (an Excel macro) [58].

### 3.3. Estimation of Diversity Indices

All data analyses were performed in R version 4.0.3 [59]. The binned dataset was first clone corrected with *poppr* version 2.8.6 [60]. The two USNA samples were genetically identical at the investigated loci, so one of these samples was eliminated from further consideration, resulting in a 19-sample dataset with unique multi-locus genotypes. The 19-sample dataset was then used to calculate various population diversity indices, including the following: number of alleles, percent missing data, Shannon’s diversity index, expected and observed heterozygosity. Indices were calculated using *poppr* and *hierfstat* version 0.5–7 [61]. The package *poppr* was also used in calculations to test if pairs of loci were in linkage disequilibrium. Calculations were performed with the standardized index of association (${\bar{r}}_{d}$), which takes the number of loci used into account as opposed to the index of association (I_A_) [47].

## 4. Conclusions

The 15 gSSR markers developed from *V. farreri* in this study are likely distributed throughout the genome, are polymorphic, and thus informative, and useful for estimating genetic diversity. The polymorphic loci will be beneficial in more advanced studies of *V. farreri* and informative in cross-transfer studies involving many *Viburnum* species across all clades as well as closely related genera.

## Figures and Tables

**Table 1:** Characteristics of 15 gSSR loci developed from *Viburnum farreri.*

Locus	GenBank #	Primer Sequences	Repeat Motif	Allele Size Range (bp)	N	Missing (%)	H’	H_o_	H_e_
**VF20_01**	MW326735	F: ACGATAAATGTGTATGCTCGC	[AT]_6_	203–205	2	0.00	0.66	0.00	0.48
		R: AACCCGGGAAGAAAGGTTACC							
**VF20_02**	MW326736	F: GAACCCTTTGAACACATGGCC	[AC]_13_	280–300	6	0.00	1.61	0.16	0.80
		R: CCAAGAAGCTTCGAAACTAGTTCC							
**VF20_03**	MW326737	F: AGCAATGTTCTAGGTCAGGGC	[GA]_6_	177–197	7	0.00	1.24	0.26	0.62
		R: CGATTTGCCCTAATCTTAGCGC							
**VF20_05**	MW326738	F: TGAAATGCAGACTGAAACGC	[AT]_7_	290–315	6	0.00	1.43	0.00	0.72
		R: GTTTGGTTCACGTCTGGTTGG							
**VF20_14**	MW326739	F: GGTTCACTGTTCATATGAATGATGC	[TC]_7_	218–245	6	5.26	1.51	0.11	0.75
		R: ATAAAGAAGTGCCACCCGTCC							
**VF20_17**	MW326740	F: GATGGTGCCAACTGATGAAGC	[AT]_12_	366-385	8	5.26	1.62	0.83	0.74
		R: GACTTCTAGGAGGTTGGTGCC							
**VF20_20**	MW326741	F: AATGCTCAAATTGCTTACGC	[TA]_9_	116–130	5	0.00	1.47	0.42	0.76
		R: TCTTAGAGCCTTGGATACTCCG							
**VF20_21**	MW326742	F: TAGATGCCTTGTTGTTGTTGC	[TAT]_7_	176–196	6	0.00	1.33	0.37	0.67
		R: CAAACGTGATTGCTGGATGGG							
**VF20_22**	MW326743	F: TCAATCAGAGCCTTGTTTGTGC	[GTA]_6_	117–119	2	0.00	0.58	0.00	0.40
		R: ATTGTTTGTTGCAGCTTTGGC							
**VF20_24**	MW326744	F: GGAGGAGATATGAGTGGGTTGG	[TAT]_6_	358–392	6	15.79	1.20	0.12	0.59
		R: AGATGATGATGATGAGTGTACC							
**VF20_37**	MW326745	F: GTTGACAGCGTTATGAAATTGG	[AAAT]_4_	390–395	2	5.26	0.69	0.11	0.51
		R: CCATAACCTAGGATCCTTGAGC							
**VF20_41**	MW326746	F: TCAGGTTGGCTCATGATACCG	[TCCC]_4_	391–394	2	21.05	0.69	0.00	0.51
		R: ATGGAACCACTACAACCAACC							
**VF20_43**	MW326747	F: TTCACGGTGAGTCAAGGAACC	[TTTA]_5_	284–314	3	5.26	0.85	0.11	0.55
		R: ATTGAAATGCAAGGGTCGACC							
**VF20_44**	MW326748	F: ATTTGACAACAACCCTACGCG	[TCTT]_4_	363–376	4	0.00	1.37	0.84	0.76
		R: GGCATGAGTAGGATGAAATGTTGG							
**VF20_46**	MW326749	F: ACATGCTTTGCACATGAAGGG	[TTTA]_4_	150–182	2	0.00	0.21	0.11	0.10
		R: AACAACCCGAACCTGACTTGC							
**Mean**					4.47	3.86	1.10	0.23	0.60

N = number of alleles; Missing (%) = percent of primers that did not amplify; H’ = Shannon’s diversity index; H_e_ = expected heterozygosity; H_o_ = observed heterozygosity.

**Table 2:** Twenty-two *Viburnum farreri* specimens included in this study.

Species/Cultivars Analyzed	Specimen Origin/Accession Number ^a^	Provenance	Year Collected
*V. farreri*	MtA 785	no record	no record
*V. farreri*	UWBG 1190-49	Garden Origin	no record
*V. farreri* ^b^	NA 0111167	Garden Origin	1938
*V. farreri* ^b^	NA 0111164	Garden Origin	1941
*V. farreri*	MA 314-49*2	Garden Origin	1949
*V. farreri*	NA 0111168	Garden Origin	1966
*V. farreri*	NA 0111169	Garden Origin	1966
*V. farreri*	MA 533-70*2	Garden Origin	1970
*V. farreri*	MA 398-83*1	Garden Origin	1983
*V. farreri*	NA 0111163	Garden Origin	1985
*V. farreri*	USNA 59728-H	Garden Origin	1987
*V. farreri*	USNA 59728-J	Garden Origin	1987
*V. farreri*	NA 0111166	Garden Origin	1987
*V. farreri*	MA 915-2005*2	Known wild origin	2005
*V. farreri*	NA 0052257	Garden Origin	2007
*V. farreri* ‘Album’	MA 1036-40*1	Garden Origin	1940
*V. farreri* ‘Candidissimum’	UWBG 1052-52	Garden Origin	no record
*V. farreri* ‘Candidissimum’	MtA 200704033 5664	no record	2007
*V. farreri* ‘KLMW’	MA 120-2012*1	Garden Origin	2012
*V. farreri* ‘Nanum’	MA 252-2002*1	Garden Origin	2002
*V. farreri* ‘Nanum’	AA 293-2003*C	Garden Origin	2003
*V. farreri* ‘Nanum’ ^c^	NCSU 2020-063	Garden Origin	2020

^a^ Sample sources: MA = The Morton Arboretum, Lisle, IL; MtA = Mt. Airy Arboretum, Cincinnati, OH; AA = Arnold Arboretum, Boston, MA; NA = U.S. National Arboretum Herbarium, Washington, D.C.; UWBG = University of Washington Botanical Garden, Seattle, WA; USNA = U.S. National Arboretum, Washington, D.C. ^b^ Samples were excluded from analysis due to low amplification rates. ^c^ Sample used for NGS sequencing and gSSR development.

## Data Availability

The data presented in this study are openly available at NCBI Bioproject PRJNA706016.
